# Phase Ib study evaluating a self-adjuvanted mRNA cancer vaccine (RNActive®) combined with local radiation as consolidation and maintenance treatment for patients with stage IV non-small cell lung cancer

**DOI:** 10.1186/1471-2407-14-748

**Published:** 2014-10-06

**Authors:** Martin Sebastian, Alexandros Papachristofilou, Christian Weiss, Martin Früh, Richard Cathomas, Wolfgang Hilbe, Thomas Wehler, Gerd Rippin, Sven D Koch, Birgit Scheel, Mariola Fotin-Mleczek, Regina Heidenreich, Karl-Josef Kallen, Ulrike Gnad-Vogt, Alfred Zippelius

**Affiliations:** Department of Hematology and Oncology, Johann-Wolfgang-Goethe-Universität, Frankfurt, Germany; Department of Radiation Oncology, University Hospital Basel, Basel, Switzerland; Department of Radiation Therapy and Oncology, Goethe University, Frankfurt am Main, Germany; Department of Medical Oncology and Hematology, Kantonsspital St Gallen, St Gallen, Switzerland; Medical Oncology, Kantonsspital Graubünden, Chur, Switzerland; Department of General Internal Medicine, Oncology, University Hospital, Innsbruck, Austria; Third Department of Internal Medicine, University Hospital Mainz, Mainz, Germany; Rippin Consulting, Solingen, Germany; CureVac GmbH, Tübingen, Germany; Department of Oncology, University Hospital Basel, Petersgraben 4, CH - 4031 Basel, Switzerland

**Keywords:** Non-small cell lung cancer, CV9202, mRNA vaccine, RNActive, Local radiotherapy

## Abstract

**Background:**

Advanced non-small cell lung cancer (NSCLC) represents a significant unmet medical need. Despite advances with targeted therapies in a small subset of patients, fewer than 20% of patients survive for more than two years after diagnosis. Cancer vaccines are a promising therapeutic approach that offers the potential for durable responses through the engagement of the patient’s own immune system. CV9202 is a self-adjuvanting mRNA vaccine that targets six antigens commonly expressed in NSCLC (NY-ESO-1, MAGEC1, MAGEC2, 5 T4, survivin, and MUC1).

**Methods/Design:**

The trial will assess the safety and tolerability of CV9202 vaccination combined with local radiation designed to enhance immune responses and will include patients with stage IV NSCLC and a response or stable disease after first-line chemotherapy or therapy with an EGFR tyrosine kinase inhibitor. Three histological and molecular subtypes of NSCLC will be investigated (squamous and non-squamous cell with/without EGFR mutations). All patients will receive two initial vaccinations with CV9202 prior to local radiotherapy (5 GY per day for four successive days) followed by further vaccinations until disease progression. The primary endpoint of the study is the number of patients experiencing Grade >3 treatment-related adverse events. Pharmacodynamic analyses include the assessment of immune responses to the antigens encoded by CV9202 and others not included in the panel (antigen spreading) and standard efficacy assessments.

**Discussion:**

RNActive self-adjuvanted mRNA vaccines offer the potential for simultaneously inducing immune responses to a wide panel of antigens commonly expressed in tumors. This trial will assess the feasibility of this approach in combination with local radiotherapy in NSCLC patients.

**Trial registration:**

Clinicaltrials.gov: NCT01915524/EudraCT No.: 2012-004230-41

## Background

Lung cancer is the leading cause of cancer-related mortality in both men and women and the incidence of the disease is increasing globally [1]. Approximately 85% of patients with lung cancer have non-small cell lung cancer (NSCLC) and 40% of these individuals will have stage IV metastatic disease at diagnosis [2]. These patients have a particularly poor prognosis and represent a significant unmet medical need. The selection of appropriate therapy is determined by the histological and molecular subtype of the disease. Four to six cycles of a non-pemetrexed-containing platinum-based combination chemotherapy is the recommended first-line therapy for fit patients with squamous cell histology, whereas in patients with non-squamous cell histology, a chemotherapy combination of pemetrexed and a platinum-based chemotherapy is a well-accepted standard of care [3]. However, the survival benefit with these regimens is modest, with a median overall survival (OS) of around 10–14 months [3–6]. Patients with non-squamous tumors harboring activating epidermal growth factor receptor (EGFR) mutations achieve impressive response rates when treated with the EGFR tyrosine kinase inhibitors (TKIs) erlotinib, gefitinib and afatinib [7–11]. These targeted therapies achieve a median OS of approximately 2–3 years. Similarly, patients with tumors containing anaplastic lymphoma receptor tyrosine kinase (ALK) fusion oncogenes achieve higher response rates when treated with the ALK inhibitor crizotinib compared with chemotherapy in patients who have received prior chemotherapy [12]. However, the effect of targeted therapies is limited due to the inevitable development of resistance, and patients with these driver mutations cannot yet be cured [13]. Therefore, novel and well-tolerated therapies that improve outcomes for all patients with NSCLC are clearly needed.

Several types of active immunotherapy are currently under investigation in NSCLC, including antibodies designed to overcome inhibitory immune signals such as ipilimumab, which targets the inhibitory CTLA-4 receptor on cytotoxic T-lymphocytes [14], and antibodies that target the inhibitory programmed death 1 receptor and its ligand [15]. Stimulating the patient’s own immune system to attack malignant cells through therapeutic vaccination against cancer-associated antigens is another promising approach [16]. Phase II studies have shown that cancer vaccines are well tolerated in patients with advanced NSCLC and several therapies have now entered or completed phase III trials (Table 1) [17–24]. RNActive® (CureVac GmbH, Germany) self-adjuvanted mRNA vaccines are a novel technology in which the mRNA sequences are optimized to enhance antigen expression by up to 4-5 orders of magnitude [25, 26]. The vaccine consists of two components: free mRNA and mRNA complexed with the cationic protein protamine. This complexed part of the vaccine has been shown to activate the immune system by involvement of toll-like receptor (TLR) 7 [25]. This results in a strong and balanced immune response comprising both humoral and cellular responses against the encoded antigens [25]. RNActive vaccines encoding different cancer antigens have been investigated in two phase I/IIA trials in patients with advanced prostate cancer and NSCLC where they were well-tolerated and induced antigen-specific cellular and humoral immune responses [23, 27].

**Table 1:** **Cancer vaccination approaches investigated in NSCLC**

Vaccine	Target(s)	Indication	Key results
CIMAVax EGF (recombinant peptide vaccine)	EGF	Pre-treated stage IIIB/IV NSCLC (Phase II, N = 80) [17]	Improved OS in younger (<60 years of age) patients compared with BSC alone
BLP25 (Stimuvax®; lysosomal peptide vaccine)	MUC1	Pre-treated stage IIIB/IV NSCLC (Phase II, N = 88) [18]	Improved QoL compared with BSC
		Pretreated, unresectable stage III NSCLC (Phase III, N = 1513) [19]	Improved survival in a prespecified stratum of >800 patients with locoregional stage IIIB disease treated with concomitant chemoradiation
TG4010 (recombinant vaccinia virus)	MUC1/IL-2	MUC1-positive stage IIIB or IV NSCLC (Phase IIb, N = 148) [20]	Trend for improved PFS compared with chemotherapy alone
Recombinant fusion protein of MAGEA3 and *H influenzae* protein D	MAGEA3	Completely resected MAGEA3-positive stage IB to II NSCLC (Phase II, N = 182) [21]	All patients receiving the active treatment showed a humoral immune response to the MAGEA3 antigen
		MAGEA3-positive stages IB, II and IIIA NSCLC (Phase III, N = 2278) [22]	Did not extend DFS compared with placebo. Trial continuing
CV9201 self-adjuvanted mRNA vaccine	MAGEC1, MAGEC2, NY-ESO-1, survivin, 5 T4	Pre-treated stage IIIB/IV NSCLC (Phase I/IIa, N = 46) [23]	Antigen-specific immune responses against ≥1 antigen were induced in 65% of patients
Belagenpumatucel-L (Lucanix®)	TGF-β2	Pre-treated stage IIIA/IIIB/IV NSCLC (Phase III, N = 532) [24]	Improved OS in subset of patients randomized within 12 weeks of completion of prior chemotherapy

BSC, best supportive care; DFS, disease-free survival; EGF, epidermal growth factor; MUC1, mucin 1, cell surface associated; IL-2, interleukin-2; MAGEA3/C1/C2, melanoma antigen family A3/C1/C2; NSCLC, non-small cell lung cancer; NY-ESO-1, New York esophageal squamous cell carcinoma 1; OS, overall survival; PFS, progression-free survival; QoL, quality of life.

The CV9202 vaccine consists of six mRNAs that code for six different NSCLC-associated antigens (Table 2). Three of these mRNAs encode cancer testes antigens (NY-ESO-1, MAGEC1, and MAGEC2) which are normally only expressed in male germ cells but are often also expressed in tumors including NSCLC, making them an attractive target for cancer vaccines [28]. NY-ESO-1 is one of the most immunogenic tumor antigens defined to date and its expression in tumor may correlate with poor survival [29, 30]. The other mRNAs encode 5 T4, survivin and MUC1. The trophoblast glycoprotein 5 T4 is expressed on undifferentiated, tumor-initiating cells in NSCLC and predicts poor clinical outcome [31]. Survivin is commonly overexpressed in NSCLC and is associated with reduced survival [32]. With the inclusion of MUC1, which is overexpressed and abnormally glycosylated on almost all adenocarcinoma epithelia cells [33], CV9202 also targets an antigen that has shown promising clinical effects as a vaccination target in several clinical trials in NSCLC patients [18–20].

**Table 2:** **Composition of CV9202**

Name	Gene symbol (other names)	mRNA length
New York esophageal squamous cell carcinoma 1	NY-ESO-1	760 bases
	(CTAG1B)	
Melanoma antigen family C 1	MAGEC1	1813 bases
	(CT7)	
Melanoma antigen family C 2	MAGEC2	1339 bases
	(CT10/HCA587)	
Baculoviral IAP repeat-containing 5	BIRC5	646 bases
	(survivin/API4)	
Trophoblast glycoprotein	TPBG	1480 bases
	(5 T4/5 T4-AG/M6P1)	
Mucin 1, cell surface associated	MUC1	1885 bases
	(PEM)	

API4, apoptosis inhibitor 4; CT7/10, Cancer/testis antigen 7/10; CTAG1b, cancer/testis antigen 1B; HCA587, Hepatocellular Cancer Antigen 587; PEM, polymorphic epithelial mucin.

While radiotherapy was historically assumed to be immunosuppressive, the release of tumor cell antigens into the tumor microenvironment following radiotherapy-induced cell death actually represents a form of immunogenic cell death that can stimulate a tumor-specific immune response [34, 35]. This is best supported by the observation of tumor response in metastatic lesions after the irradiation of the primary tumor, the so called ‘abscopal effect’ [36]. A recent clinical case report describes a regression of non-irradiated metastases in a patient with metastatic melanoma who had disease progression during treatment with ipilimumab after irradiation of a mediastinal metastasis with 3 × 9 GY [37]. Immunogenic cell death is characterized by cell surface translocation of calreticulin and extracellular release of ATP and the high-mobility-group box 1 (HMGB1) protein [34, 38]. HMGB1 binds to TLR4 expressed on dendritic cells and promotes the cross-presentation of tumor-antigens between dendritic cells and T cells, an integral part of the immune response [39, 40]. Radiotherapy also induces the release of pro-inflammatory components into the tumor microenvironment [41], and upregulates MHC class 1 molecules on the tumor cells [42] which further potentiate this response.

Synergism between vaccination or CTLA4 blockade and radiotherapy has been reported in preclinical models for both single-dose radiation and fractionated regimens [43–45]. The synergy between immunotherapies and radiotherapy may be greater when radiation is given as a fractionated regimen compared with single dose radiotherapy – complete primary tumor regression was seen in mice breast cancer models treated with an anti-CTL4 antibody and fractionated (3 × 8 GY) radiotherapy, but not in mice treated with antibody alone or antibody with single-dose radiotherapy [44]. Furthermore, an abscopal effect was seen only in the mice treated with the fractionated radio-immunotherapy combination [44]. Fractionated regimens may also achieve an optimal balance between a high level of T cell cross-priming with low induction of Treg cells [46]. Synergism between mRNA vaccination and fractionated local radiation was seen in C57BL/6 mice bearing subcutaneous immunogenic E.G7-OVA tumors (treated with 3–4 × 2 GY) or low immunogenic Lewis lung carcinoma cells (treated with fractions of 3 × 12 GY) [CureVac, [47]. In the latter model, combination treatment resulted in an increased infiltration of both innate (CD11c dendritic cells, CD11b + myeloid cells, NK, and NK T cells) and adaptive (CD4+ and CD8+ T cells) tumor-infiltrating immune cells. Given these observations, local radiation of individual tumor sites in patients with metastatic cancer may be an effective way to enhance the systemic antitumor effect of a cancer vaccine.

The aim of this study is to assess the safety and tolerability of the CV9202 vaccine in combination with local radiation in patients with stage IV NSCLC who achieved a response or stable disease after first-line therapy, either with chemotherapy or EGFR TKIs. We have selected a regimen of 4 × 5 GY, a well-established palliative radiation regimen that can be safely applied to metastatic lesions in the lung, bone, and soft tissue [48].

## Methods/Design

### Study design

Study CV-9202-006 (Trial registration number: NCT01915524) is an exploratory, open-label multicenter phase Ib trial of RNActive-derived cancer vaccine and local radiation as consolidation and maintenance treatment in patients with stage IV NSCLC achieving a response or stable disease after first-line therapy (chemotherapy or therapy with an EGFR tyrosine kinase inhibitor). The study will be conducted according to good clinical practice and the Declaration of Helsinki and in keeping with local regulations. Written informed consent will be obtained from all patients before any study-related activities are conducted. This study was approved by the ethics committees of University Hospital Basel, Kantonsspital St Gallen, Kantonsspital Winterthur, and Kantonsspital Graubünden in Switzerland; University Hospital Mainz and University Hospital Frankfurt in Germany; and University Hospital Innsbruck in Austria.

Patient recruitment is currently underway in seven European centers and accrual is expected to take approximately 18 months. The inclusion and exclusion criteria are listed in Table 3. To investigate the activity of CV9202 across the spectrum of NSCLC, patients will be enrolled into one of three study arms based on the histological and molecular subtype of NSCLC:

**Table 3:** **Inclusion and exclusion criteria**

Criteria	Details
**Inclusion criteria**	• Histologically or cytologically-confirmed metastatic NSCLC (stage IV)
	• ≥18 years of age
	• Presence of at least one tumor lesion ≥ 2 cm in size that is eligible for radiation and at least one additional measurable tumor lesion according to RECIST Ver 1.1
	• ECOG performance status 0 to 1
	• Adequate organ function: hemoglobin ≥95 g/L, platelet count ≥75000/μL, white blood cell count ≥2000/μL, absolute neutrophil count ≥1000/μL, lymphocyte count ≥0.8 × 10^9^/L, ALT and AST ≤2.5 times ULN in patients without liver metastases and ≤5 times ULN in patients with liver metastases, serum creatinine ≤2 mg/dL, creatinine clearance ≥45 mL/min according to MDRD formula
**Exclusion criteria**	• Previous active immunotherapy for NSCLC (including vaccination, therapy with anti-CTLA4 antibodies)
	• Treatment with any investigational product in the 4 weeks prior to study entry
	• Need for immunosuppressive treatment
	• Active skin disease not allowing intradermal injections into areas of healthy skin for vaccine injection (for stratum 3 patients: persisting grade 3 skin rash at time of enrollment)
	• Inadequate lung function dependent on the intended tumor volume and location to be irradiated (for patient planned to undergo radiation of thoracic lesions)
	• Prior splenectomy or allogeneic bone marrow transplantation; history of pneumonitis, encephalitis or multiple sclerosis; active inflammatory conditions or autoimmune disorders (except for vitiligo, diabetes mellitus type 1 or autoimmune thyroiditis requiring hormone replacement only), primary or secondary immune deficiency, seropositivity for HIV, HBV, HCV or any other infection requiring anti-infection therapy; known brain metastases (except for stable metastases being treated with stereotactic radiation or surgery)
	• Uncontrolled medical condition considered as high risk for the treatment with an investigational drug, unstable angina pectoris/myocardial infarction within the previous 6 months, significant cardiac arrhythmia, stroke or transient ischemic attack within the previous 6 months, severe hypertension according to WHO criteria, uncontrolled systolic blood pressure ≥180 mmHg
	• Estimated life expectancy ≤3 months
	• Unable to consent or comply with protocol
	• Allergies to any components of the study drug
	• Pregnancy or breast feeding
	• Concurrent or planned major surgery or likelihood of requiring treatment with drugs not permitted by the clinical study protocol

ALT, alanine aminotransferase; AST, aspartate aminotransferase; CTLA4, Cytotoxic T-Lymphocyte Antigen 4; ECOG, Eastern Cooperative Oncology Group; HBV/HCV, hepatitis B/C virus; HIV, human immunodeficiency virus; MDRD, Modification of Diet in Renal Disease; NSCLC, non-small cell lung cancer; RECIST, Response Evaluation Criteria In Solid Tumors; ULN, upper limit of normal; WHO, World Health Organization.

-  
**Stratum 1:** Patients with non-squamous histology, without activating EGFR mutations, who achieved partial response (PR) or stable disease (SD) after at least four cycles of platinum- and pemetrexed-based first-line chemotherapy, and with an indication for maintenance therapy with pemetrexed.
-  
**Stratum 2:** Patients with squamous cell histology, who achieved PR or SD after at least four cycles of platinum-based and non-platinum compound first-line chemotherapy.
-  
**Stratum 3:** Patients with non-squamous histology and an activating EGFR mutation, who achieved PR after up to six months of treatment with an EGFR TKI.

### Study endpoints

The primary endpoint of the study is to determine the number of patients who experience grade 3 and above treatment-related adverse events (AEs) according to NCI-CTCAE, version 4.0 criteria. Secondary endpoints include determining the incidence of standard clinical trial clinical and laboratory assessments and evaluating the cellular and humoural immune responses to antigens encoded by the six vaccine mRNA components. Furthermore, the presence of humoral immune responses to a panel of antigens not covered by the vaccine will be evaluated to investigate the potential broadening of immune responses (‘antigen spreading’) following treatment.

Clinical response according to Response Evaluation Criteria in Solid Tumors (RECIST) criteria (version 1.1) and assessment of PFS, time to start of second-line cancer treatment, response to second-line cancer treatment, and OS are secondary efficacy endpoints of the study.

### Treatment overview

**Figure 1:**
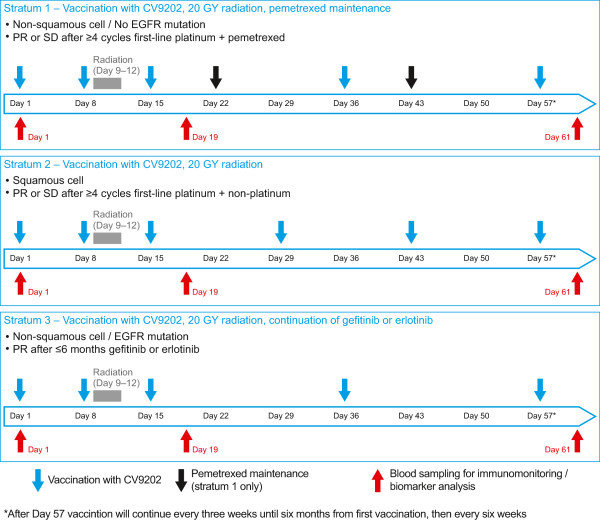
**Study design and treatment schedule.** EGFR, epidermal growth factor receptor; PR, partial response; SD, stable disease.

Patients will start screening two weeks after Day 1 of the last cycle of their first-line chemotherapy (strata 1 and 2) or within six months of starting treatment with an EGFR TKI (erlotinib or gefitinib) (stratum 3). Patients will be vaccinated twice (Day 1 and Day 8) with CV9202 before starting radiotherapy on the following day (Day 9; Figure 1). Each of the six components of CV9202 will be administered individually as two intradermal injections (to the inner part of the upper arms or thighs respectively) for a total of 12 injections distributed over the four limbs. A total dose of 1920 μg mRNA (six compounds × 320 μg mRNA per compound) will be administered per vaccination time point.

Patients with non-squamous histology will receive five vaccinations until Day 57. As the PFS for patients with squamous histology is expected to be lower than for patients with non-squamous histology, individuals recruited to strata 2 will receive a more intense vaccination schedule with six vaccinations until Day 57. A similar vaccination schedule has also been applied in other trials testing monotherapy with RNActive vaccines [23]. Preclinical data indicated that more frequent vaccinations enhance the generation of the antigen specific immune responses which supports the use of the more intensive schedule in this stratum [CureVac, data on file]. Vaccine will be administered until disease progression and the need to start a subsequent systemic second-line treatment, or occurrence of unacceptable toxicity requiring treatment discontinuation. It is anticipated that approximately six to eight vaccinations will be administered in strata 1 and 2 (based on an expected median PFS time of 2–4 months in patients with SD after the end of first-line combination chemotherapy [3]), with more vaccinations anticipated for patients in stratum 3 who have an expected median PFS of at least 9–10 months from the time of start of treatment with an EGFR TKIs [7–9].

Radiotherapy will be administered in four daily fractions of 5 GY from Day 9 to 12 (Figure 1). Lesions that are eligible for radiation are those that measure ≥2 cm in the longest diameter for lymph node lesions or ≥1 cm for non-lymph node lesions. Eligible lesions will be selected according to the following hierarchy: bone metastases (first preference); lymph nodes in the paraclavicular, axillary, or cervical regions; skin or subcutaneous metastases; and thoracic lesions (only for strata 1 and 2 patients). A treatment-planning CT scan will be used to define the macroscopic volume of the selected lesion (gross tumor volume; GTV), the clinical target volume (CTV; which includes the GTV with the surrounding tissue, where microscopic tumor involvement is highly probable), and the planning target volume (PTV; which includes the CTV with a safety margin for positioning error and dose).

Pemetrexed maintenance treatment (stratum 1) and the EGFR TKIs erlotinib or gefitinib (stratum 3) will be administered in accordance with the product label. Preclinical studies have shown that combining pemetrexed, gefitinib, or erlotinib with RNActive vaccination does not negatively affect the immune response induced by RNActive [CureVac, data on file]. Vaccination in stratum 1 will be administered 4–7 days before each scheduled dose of pemetrexed to provide an interval of at least two days between vaccination and dexamethasone, which is given as premedication one day prior to each dose of pemetrexed.

### Assessments

Standard clinical and laboratory safety assessments will be performed throughout the follow-up period with all AEs graded according to NCI-CTCAE version 4.0. Radiological tumor assessment will be performed every six weeks until end of treatment, as recommended for patients with stage IV NSCLC [49]. In selected patients, pre- and post-treatment tumor biopsies of non-irradiated lesions will be collected to evaluate whether the study treatment induces changes of immune infiltrates in the tumor and immune signature by gene expression analysis. Blood samples (peripheral blood mononuclear cells and serum) for the assessment of humoral and cellular immune responses against the antigens encoded by CV9202 as well as humoral responses to additional cancer antigens to evaluate antigen spreading will be collected at baseline (Day 1), Day 19, and Day 61. Additional blood samples for biomarker assessments after the Day 61 visit will be collected every three months. Antibody responses against antigens covered by the vaccine and non-vaccination antigens will be tested using ELISA or a bead-based assay. The quality and quantity of preexisting and vaccination-induced cellular immune responses (antigen-specific T lymphocytes) to the vaccine will be assessed by multifactorial intracellular cytokine staining, measuring CD107a, IL-2, IFN-gamma and TNF-alpha production by CD4+ and CD8 + T cells, and IFN-gamma ELISpot assay. Validated protocols (SOPs) and assay-specific response criteria are in accordance with harmonization panels and guidelines of the Immunoguiding Program of the Association for Cancer Immunotherapy (CPI/CIMT) and the Cancer Immunotherapy Consortium of the Cancer Research Institute (CIC/CRI) [50–56]. Furthermore, a detailed phenotypic analysis of blood immune cells (B cells, dendritic cells, myeloid-derived suppressor cells, macrophages, NK cells and T cells) will be performed by polychromatic flow cytometry. Cytokine/chemokine profiling in the serum samples of patients will be analyzed by cytometric bead assay.

After the first six patients in a given stratum have completed radiation, received at least three vaccinations, and have been monitored for toxicity up to Day 43, recruitment will be interrupted and the safety will be reviewed by the independent DSMB. If two or more patients experience treatment-related grade ≥3 AEs, enrollment in the respective stratum will be suspended. If the DSMB will approve further recruitment, 2–14 additional patients will be recruited per stratum until a total number of 36 patients have been included. An additional interim safety evaluation assessing the safety of radiation of thoracic lesions in combination with CV9202 will be performed in strata 1 and 2 after six patients have undergone radiation of a thoracic lesion and been evaluated for signs of radiation pneumonitis up to Day 57. If any patient experiences grade ≥3 radiation pneumonitis, no further patients will be treated with radiation of thoracic lesions.

### Statistical considerations

The planned sample size of 36 patients was chosen based on previous observations with predecessor vaccines of CV9202, which indicated that a minimum of eight patients per stratum would be required to evaluate the frequency of immune responses. The main statistical analysis is anticipated six months after enrollment of the last patient. Safety evaluations will be performed for all patients receiving at least one dose of study drug (safety analysis set) and efficacy analyses will be performed in patients treated as per-protocol. Standard measures will be used to summarize continuous (mean, standard deviation, and median) and categorical variables (frequencies and percentages). Time-to-event variables will be analyzed descriptively by the Kaplan–Meier method and probabilities calculated for specific timepoints (e.g. 12 months or 24 months). All endpoints will be evaluated individually for the different strata and safety and immune-related endpoints will be analyzed overall. After completion of study treatment, all patients will be followed up for survival every three months until death, withdrawal of informed consent for follow-up, or loss to follow-up. The follow-up period for all patients will end 18 months after start of treatment of the last patient enrolled.

## Discussion

Despite significant improvements in survival for patients with stage IV NSCLC over the last few decades, the outlook for these patients remains bleak. Survival rates achieved with conventional chemotherapy combinations have plateaued and median survival in patients with molecular alteration that can be targeted with novel drugs is only around 2–3 years [7–9]. Though these targeted therapies achieve high response rates with prolongation of PFS, new and well-tolerated therapies are urgently required. Recruiting the patient’s own immune system into the therapeutic process through the use of cancer vaccines targeted against specific cancer associated antigens is a promising approach that offers the potential to change the course of the disease and offer durable and long-lasting responses [15, 57].

Study CV-9202-006 will evaluate the safety and tolerability of the RNActive CV9202 vaccine in combination with radiotherapy. A phase I/IIa trial with a similar mRNA-based vaccine (CV9201; which contains five of the six antigens in CV9202) in 46 patients with stage III/IVB NSCLC showed that vaccination was well tolerated and induced immune responses [23]. Patients received a maximum total mRNA dose per application of 1600 μg (320 μg of each individual compound) application and no dose-limiting toxicities or serious AEs were observed; only three (7%) patients experienced a Grade ≥3 AE that was considered potentially treatment related. Antigen-specific immune responses were seen in over two-thirds of patients and a significant shift from naïve B-cells to pre-germinal center B-cells was detected in patients after vaccination. Importantly, Treg cell counts did not increase during treatment. The addition of another mRNA compound encoding the MUC1 antigen in CV9202 will correspond to a 20% increase in mRNA amount per vaccination; however, based on the dose-escalation experience in the phase I/IIa trial with CV9104 it is not expected that the dose of 1920 μg of mRNA in the current trial will significantly change the safety profile. Furthermore, vaccination with MUC1 alone appears to be well tolerated [18–20].

Clinical experience with radiotherapy-vaccine combinations to date is extremely limited. Thirty patients with localized prostate cancer received a prostate specific antigen (PSA)-containing poxviral vaccine with radiotherapy in a randomized phase II trial [58]. This combination was well tolerated and 13/17 patients treated with the combination had increased levels of PSA-specific T cells compared with none of the patients receiving radiotherapy alone. Low-dose radiotherapy administered concomitant with a vector-based carcinoembryonic antigen vaccine was also well tolerated [59]. In the recent phase III trial of the MUC1 vaccine BLP25 in patients with NSCLC, median OS in the total cohort did not significantly differ between patients randomized to the vaccine or placebo [19]. However, a clinically meaningful prolongation of OS was observed in the predefined subgroup of patients treated with vaccine and concurrent chemo-radiotherapy compared with placebo and radiotherapy (30.8 vs 20.6 months, respectively; p = 0.016), whereas the subgroup of patients receiving a sequential chemo-radiotherapy did not show a survival difference [19].

As a RNA-based vaccine, CV9202 features several advantages over other approaches, including vaccination with peptides, DNA-based vaccines or viral vaccines. Peptides bind only to certain major histocompatibility complexes and are therefore only applicable for patients with certain HLA genotypes; in contrast, there is no such restriction for RNA-based vaccines, because full proteins are encoded. In contrast to DNA-based vaccines, RNA-based vaccines do not need to cross the nuclear membrane to be active and, importantly, in the absence of reverse transcriptase, RNA cannot be integrated into the genome. Viral-based vaccines may lead to an undesirable immunodominant reaction to the foreign immunogenic virus material, which could override immune responses against the vaccination antigen. There is no such risk with RNA-based vaccines. An additional advantage of RNActive vaccines is the potential to encode a variety of cancer antigens, allowing the induction of an immune response against multiple antigens, which limits the risk of tumor escape by antigen loss and induces relevant immune responses in more patients with different antigen expression patterns. CV9202 targets three highly tumor-specific cancer testis antigens which are expressed in up to 30% of NSCLC tumors [60, 61]. In addition, the antigens survivin, 5 T4 and MUC1 are targeted, which are all expressed in >90% of NSCLC samples and detected at low levels in healthy tissues [31–33]. This composition should maximize the chance that an individual patient’s tumor will express several of the encoded antigens and might therefore benefit from vaccination.

One strength of the design of study CV-9202-006 is that it will investigate the vaccine-radiotherapy combination in three histological/molecular subtypes of NSCLC, including patients with squamous cell carcinoma histology who have a particular need for more effective therapies. The median OS in patients with EGFR-mutant advanced NSCLC (represented in strata 3) is about 2–3 years [7–9]; therefore, these patients will have received relatively long-term treatment with EGFR TKIs and achieve a relatively long progression-free period (estimated at 10 months). This offers a window of opportunity for testing immunotherapeutic approaches in NSCLC in these patients, as the vaccine has several months to induce an immune response while the tumor growth is still controlled by the TKI. A second strength of the study design is that the inclusion/exclusion criteria do not have an upper age restriction; therefore, the patient population is likely to be representative of the general population of patients with lung cancer which has a median age at diagnosis of approximately 70 years [1].

To date, 19 patients have been recruited to study CV-9202-006 and recruitment is expected to be complete by the end of 2014. Interim safety analysis of the first six patients in strata 1 and 2, as well as the first six patients treated with thoracic radiation, are completed and the DSMB has approved further recruitment since there were no safety concerns.

### Study sites

This study is to be conducted at the following sites:

-  University of Basel, Switzerland
-  Johann-Wolfgang-Goethe-Universität, Frankfurt, Germany
-  Kantonsspital St Gallen, Switzerland
-  Kantonsspital Graubünden, Chur, Switzerland
-  University Hospital, Innsbruck, Austria
-  University Hospital Mainz, Mainz, Germany
-  Pius-Hospital Oldenburg, Germany
-  HELIOS Klinikum Emil von Behring, Berlin-Zehlendorf, Germany
-  Augusta-Kranken-Anstalt gGmbH, Bochum, Germany
-  Klinikum Esslingen GmbH, Esslingen, Germany
-  Kliniken der Stadt Köln gGmbH, Cologne, Germany
-  Heidelberg University Hospital, Heidelberg, Germany

## Abbreviations

AE: Adverse events
ALK: Anaplastic lymphoma receptor tyrosine kinase
ALT: Alanine aminotransferase
API4: Apoptosis inhibitor 4
AST: Aspartate aminotransferase
BIRC5: Baculoviral inhibitor of apoptosis repeat-containing protein 5
BSC: Best supportive care
CT: Computer tomography
CIC/CRI: Cancer Immunotherapy Consortium of the Cancer Research Institute
CPI/CIMT: Immunoguiding Program of the Association for Cancer Immunotherapy
CT7/10: Cancer/testis antigen 7/10
CTCAE: Common toxicity criteria for adverse events
CTV: Clinical target volume
ECOG: Eastern Cooperative Oncology Group
EGF(R): Epidermal growth factor (receptor)
GTV: Gross tumor volume
GY: Gray
HBV/HCV: Hepatitis B/C virus
HCA587: Hepatocellular Cancer Antigen 587
HIV: Human immunodeficiency virus
HMGB1: High-mobility-group box 1protein
IL-2: Interleukin 2
MAGEA3/C1/C2: Melanoma antigen family A3/C1/C2
MDRD: Modification of Diet in Renal Disease
MUC1: Mucin 1, cell surface associated
NCI: National Cancer Institute
NSCLC: Non-small cell lung cancer
NY-ESO-1: New York esophageal squamous cell carcinoma 1
OS: Overall survival
PEM: Polymorphic epithelial mucin
PFS: Progression-free survival
PR: Partial response
PSA: Prostate specific antigen
PTV: Planning target volume
QoL: Quality of life
RECIST: Response Evaluation Criteria in Solid Tumors
RNA: Ribonucleic acid
SD: Stable disease
TKI: Tyrosine kinase inhibitor
TPBG: Trophoblast glycoprotein
Treg: T-regulatory cells
ULN: Upper limit of normal
WHO: World Health Organization.
